# Driver’s Head Pose and Gaze Zone Estimation Based on Multi-Zone Templates Registration and Multi-Frame Point Cloud Fusion

**DOI:** 10.3390/s22093154

**Published:** 2022-04-20

**Authors:** Yafei Wang, Guoliang Yuan, Xianping Fu

**Affiliations:** School of Information Science and Technology, Dalian Maritime University, Dalian 116026, China; wangyafei@dlmu.edu.cn (Y.W.); fxp@dlmu.edu.cn (X.F.)

**Keywords:** driving environment, head pose, ICP, point cloud, gaze zone

## Abstract

Head pose and eye gaze are vital clues for analysing a driver’s visual attention. Previous approaches achieve promising results from point clouds in constrained conditions. However, these approaches face challenges in the complex naturalistic driving scene. One of the challenges is that the collected point cloud data under non-uniform illumination and large head rotation is prone to partial facial occlusion. It causes bad transformation during failed template matching or incorrect feature extraction. In this paper, a novel estimation method is proposed for predicting accurate driver head pose and gaze zone using an RGB-D camera, with an effective point cloud fusion and registration strategy. In the fusion step, to reduce bad transformation, continuous multi-frame point clouds are registered and fused to generate a stable point cloud. In the registration step, to reduce reliance on template registration, multiple point clouds in the nearest neighbor gaze zone are utilized as a template point cloud. A coarse transformation computed by the normal distributions transform is used as the initial transformation, and updated with particle filter. A gaze zone estimator is trained by combining the head pose and eye image features, in which the head pose is predicted by point cloud registration, and the eye image features are extracted via multi-scale spare coding. Extensive experiments demonstrate that the proposed strategy achieves better results on head pose tracking, and also has a low error on gaze zone classification.

## 1. Introduction

The head pose and eye gaze are significant clues for indicating a driver’s visual attention. In the automotive context, drivers need to constantly move their head and eyes, and maintain an effective perception of the surrounding environment at all times. Monitoring head pose and eye gaze is a vital task and key component of the advanced driver assistance system. The system helps in understanding the driver’s visual attention and monitors awareness during on-road driving, especially detecting distraction or drowsiness in the driver [1,2,3]. Even in the Level 3 automated driving system, the head pose and gaze zone measure should still be needed to monitor the visual attention during takeover actions. Therefore, achieving accurate head pose estimation and gaze zone classification is crucial for the in-vehicle eye gaze tracking system.

**Figure 1:**
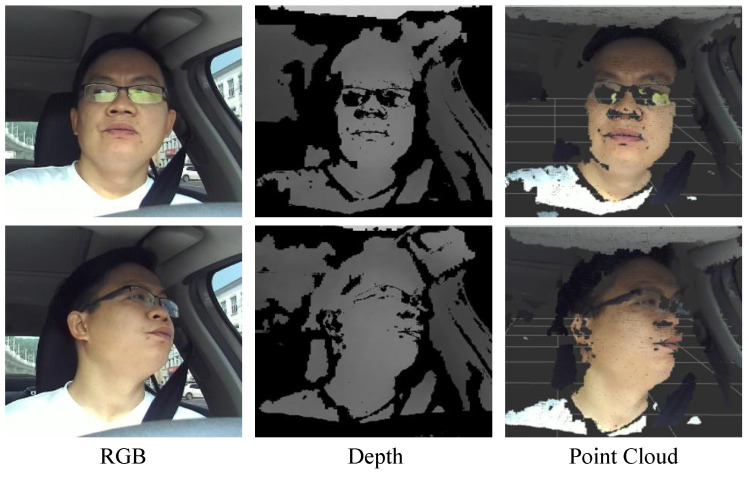
Challenges in the naturalistic driving scene.

The head pose and gaze estimation using an RGB-D camera is an active topic in recent computer vision research. By taking advantage of the depth information, the approaches based on RGB-D cameras commonly predict the head pose and gaze zone with depth appearance extraction [4,5] or point cloud template matching [6,7]. These previous works show promising results in constrained conditions. However, there is not much progress on the robust head pose and gaze zone estimation in real automotive applications [6,8,9,10,11]. Existing research cannot predict accurate results in challenging driving conditions, due to extreme facial occlusion, large head movement, and non-uniform illumination. Challenging conditions seriously affect the quality of point clouds, and can cause point cloud fragmentation. As shown in Figure 1, the depth images collected by the RGB-D camera have partial information occlusion, and result in randomly distributed empty areas, such as the nose and mouth periphery. The constructed 3D point cloud data is accordingly missing. These occlusions usually lead to bad transformation caused by inaccurate point cloud registration.

Therefore, this paper focuses on providing an effective solution for the head pose and gaze zone prediction based on point cloud registration in the real driving conditions. Differing from previous point cloud registration works [6,8,9], this paper further studies the solution to improving the efficiency of point cloud fusion, and presents a whole process for point cloud registration. To alleviate failure problems, multi-frame point cloud fusion is applied to avoid the partial missing of a single frame point cloud. To reduce the impact of the transformation initialization, coarse transformation is adopted before iterative registration by the normal distribution transformation, and is updated with a particle filter. Besides, multiple templates of the nearest neighbor gaze zone are used to accelerate the transformation. The highlights of this paper are the following:

- A novel method for estimating the driver’s head pose and gaze zone from RGB-D data is proposed based on multi-frame point cloud fusion and multi-zone point cloud registration. This method generates a stable head point cloud by revising and fusing consecutive multi-frame point clouds. It avoids information loss from the partially missing point cloud templates and source.
- An effective method for aligning the driver’s head point cloud is presented to compute the best transformation in template matching. This method reduces the impact of registration initialization by status tracking and coarse transformation. It ensures high registration accuracy for multi-zone point cloud registration.
- Extensive experiments demonstrate that the proposed method reduces the head pose estimation error, and has reliable results on the head pose estimation and gaze zone classification.

The rest of this paper is organized as follows. Section 2 briefly introduces the related driver head pose and gaze zone estimation works. In Section 3 describes the multi-zone point cloud registration for head pose tracking, the multi-frame point cloud fusion, and gaze zone estimation in detail. Section 4 shows the comparisons and evaluations of the proposed method. In Section 5, the conclusion is presented.

## 2. Related Works

### 2.1. Head Pose Estimation Using RGB-D Camera

The driver’s head pose estimation has always been a hot topic in the field of intelligent transportation system research, which is generally based on the RGB camera or the RGB camera with infrared filters. In recent years, research using point cloud data from the RGB-D camera has gradually attracted attention. More detailed surveys can be found in Refs. [12,13]. The commonly applied approaches for point cloud data or depth image are random forest [4], optimization algorithms [14], and the ICP-based method [6,7,8,9]. There have also been various attempts to approach convolutional neural networks [10,11,15,16,17]. The methods using RGB-D cameras can be divided into two types, the first one is the feature-based methods, the other is the template-matching methods.

#### 2.1.1. Feature-Based Methods

Feature-based methods usually take the depth image of the face region or eye region as the image features, build the related regressors using data-driven technology, and output continuous head pose values. Fanelli et al. [4] divided the depth image into random image depth patches, and selected the most probable result via node voting in the random forest. Saeed and Al-Hamadi [18] built an estimation model by SVM, utilizing all relevant information from RGB-D camera.

Deep learning methods have been applied in current research on depth images [15,16,19]. Aoki et al. [20] proposed PointNetLK to extract relative rigid pose information from two point clouds using PointNet [21] with the T-net module removed. They used the inverse compositional formula to calculate the Jacobian matrix of the global features of the target point cloud. The differentiable Lucas and Kanade (LK) algorithm was used to optimize the differences between global features to compute rigid transformations. Huang et al. [22] modified this network using autoencoding and point distance loss. In the pose estimation stage, the traditional optimization method was applied to calculate the Jacobian matrix and estimate the motion parameters from the features. This caused a large computational cost. There is also deep learning research progress in the driving conditions. Hu et al. [10,11] presented the first end-to-end solution of head pose estimation from point clouds using the PointNet++ network [23] with a revised set abstraction layer. Their networks captured the features by shared multilayer perceptrons, and generated the output from the last layer to compute the head status. The sampling of the deep learning-based methods do not have satisfying generalization results in the driving conditions. This may caused by the unstructured, disordered and irregular nature of the point cloud data, which is not conducive to the extension of structured methods to the point cloud.

#### 2.1.2. Template-Matching Methods

Template-matching methods classically treat the head pose problem as the rigid registration between the source point cloud and the template point cloud. The head pose value is calculated by optimizing the registration to get the best transformation. These methods are usually based on the ICP algorithm [24,25]. Padeleris et al. [14] explored the point cloud registration using a particle swarm optimization algorithm. The algorithm can be used for solving the optimal transformation. Meyer et al. [7] added a further step, combining the ICP algorithm with the particle swarm optimization algorithm. Yang et al. [26] proposed the go-ICP algorithm to find the global optimal transformation by the threshold condition. The initial space was subdivided into smaller subspaces using the octree data structure. Although this method solves the local minima problem, it is still sensitive to initialization. Pavlov et al. [27] introduced the Anderson acceleration into the ICP algorithm, and proposed two heuristic strategies to deal with the Anderson algorithm’s acceleration. The convergence speed and robustness of registration were improved.

For the head pose estimation in the driving environments, Peláez C. et al. [8] collected the head point cloud data in a fixed area, and realized the iterative solution of the head pose through the ICP algorithm. They used simple cascaded face region detection to crop the side region of the point cloud to eliminate the interference of point cloud noise. Bär et al. [9] proposed a strategy to utilize multiple templates for simultaneous point cloud registration. The point cloud templates of the left and right faces were pre-collected and merged into one template. This multiple template strategy was mainly considered the case when only a half-face was detected. To accelerate the registration process, the Newton method was used in the approach. They also extracted the gaze direction on the eye model template. Due to the strictness of the initial value, this method might be trapped in local solutions. It is worth noting that these methods all used Kinect to collect point cloud data, and an infrared projector on the camera was easily affected by non-uniform illumination. During point cloud registration under large rotation and translation conditions, the ICP often fails to obtain correct results. Wang et al. [6] tried to use the characteristics of the driving environment to improve the registration accuracy. Multiple templates for different gaze zones were initialized, and particle filter tracking was used to select the gaze zone to be registered.

The ICP algorithm has high precision and a wide application range in the driving environment. Previous methods generally focus on preventing the local optima with the initialization. These methods still suffer from severe challenges under real driving conditions, which restricts their usage [28]. One of the challenges is facial occlusion under non-uniform illumination and large head movement, which leads to unreliable point cloud data quality. In the corresponding template matching, the occlusion has a great impact on point cloud registration, and limits the accuracy of registration by the incorrect transformation. There is little progress on generalizing the robust driver head pose estimation in challenging conditions from point cloud data.

### 2.2. Driver’s Gaze Zone Estimation

Eye gaze dynamics are an important indicator of the driver’s visual attention. The majority of eye movements in the driving scenarios are accompanied by variation in head movement. Many existing works divide the gaze region in front of the driver into several gaze zones, and convert the gaze estimation into gaze zone estimation [29,30,31,32,33]. The gaze zone estimation outputs rough gaze prediction results, which is practical and applicable. Among them, some of the gaze zone estimation works studied only the head pose, other works used both head pose and eye pose. More detailed surveys can be found in Refs. [34,35].

Existing works predict the driver’s eye gaze by regressing the facial features or pupil features to the gaze location or gaze angle [30,31,36,37]. The regression models are built by recent advanced techniques. For the driver’s gaze zone estimation, the regression model is replaced by classification model. Researchers [32,33] used transfer learning to predict the driver’s gaze zone. In Refs. [38,39,40], they studied the driver’s gaze estimation by the probabilistic regression method, in which, one of the most important techniques is the Gaussian process regression. Fridman et al. [29,41] integrated face detection, facial landmark detection, eye region detection, pupil detection, and gaze zone estimation into one combined system. This kind of feature-based method had better accuracy, especially after subject-dependent calibration. Ledezma et al. [42] also explored a feature-based method to predict the driver’s gaze location. Araluce et al. [43] located eye gaze via opensource toolkit in accidental scenarios. Chiou et al. [44] monitored the driver eye gaze using sparse representation with part-based temporal face descriptors. Yang et al. [45] estimated the driver’s head pose in the facial landmarks process, and used it as concatenated features in the hidden layers of the convolutional neural networks. Their results demonstrated that the fused head pose feature benefited the estimation of gaze zone.

For gaze zone estimation using an RGB-D camera, Refs. [6,8] both used eye regions on the RGB image, and ignored the corresponding depth image. The main reason is that the depth image of the eye regions is incomplete and partially missing due to the facial occlusion. These methods were based on global features or pupil localization, and extracted the features by global representation, which cannot effectively characterize the appearance information in the eye image. Therefore, this paper used a multi-scale encoding method for the characterization of the eye images.

## 3. Proposed Method

**Figure 2:**
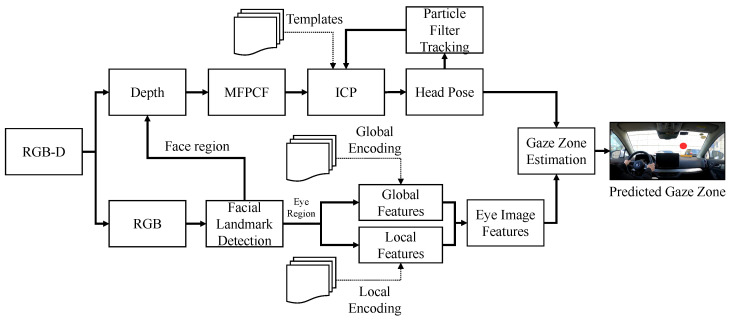
Overview of the proposed driver gaze zone estimation method.

The proposed approach has three main parts: head pose estimation, eye image feature extraction, and gaze zone estimation, as shown in Figure 2. The process of head pose estimation is on the point cloud of the face region, while the process of eye image feature extraction is on the RGB image of the eye region.

In the head pose estimation part, point cloud registration is used for predicting head pose. Supported by the multiple templates, the driver’s current gaze zone point cloud is adopted to reduce the templates accumulative error in continuous registration. This paper aligns the source point cloud and templates point cloud by iterative method. Multi-frame point cloud fusion is applied when generating the point cloud sets to complete the head point cloud. The head status is initialized and updated via particle filtering, and its accurate value is output by transformation computed in the registration.

In the eye image feature extraction part, the eye region is obtained by facial landmark detection, which is restricted within the upper face. Its image is segmented and normalized to adjust the illumination of the image. This paper uses a multi-scale sparse coding representation method for the characterization of the given images. The eye images are mapped into high-dimensional sparse encoding space. In the gaze zone estimation step, this paper does not solve the eye pose value, but uses the eye images as features. Combining the head pose vector and eye image features, the final gaze zone is predicted by a multi-class SVM classifier. In this section, each step of the proposed approach is introduced in detail.

### 3.1. Head Pose Estimation Based on Point Cloud Registration

To estimate the driver’s current head pose or head movement in the given point cloud sets, the template point cloud with known head pose is used as a reference. The face model can be regarded as a rigid structure without deformation. By calculating the transformation between the source point cloud and template point cloud, the point clouds are unified under the same coordinate system. In this paper, the transformation is defined as follows:

(1) $$T=\left[\begin{array}{cc} R & t\\ 0 & 1 \end{array}\right]$$

where R denotes the rotation matrix, $t={\left[t_{x},t_{y},t_{z}\right]}^{T}$ is the translation vector. This rotation matrix can be divided into three rotation matrices corresponding to the coordinates, which is expressed by the formula:

(2) $$\begin{array}{c} R=R_{x}\cdot R_{y}\cdot R_{z}\\ R_{x}=\left[\begin{array}{ccc} 1 & 0 & 0\\ 0 & \cos\alpha & \sin\alpha \\ 0 & -\sin\alpha & \cos\alpha \end{array}\right]\\ R_{y}=\left[\begin{array}{ccc} \cos\beta & 0 & \sin\beta \\ 0 & 1 & 0\\ -\sin\beta & 0 & \cos\beta \end{array}\right]\\ R_{z}=\left[\begin{array}{ccc} \cos\gamma & \sin\gamma & 0\\ -\sin\gamma & \cos\gamma & 0\\ 0 & 0 & 1 \end{array}\right] \end{array}$$

When the fine transformation matrix is computed via point cloud registration, the head pose can be obtained by the formula:

(3) $$\begin{array}{cc} \; & \alpha =\arctan\left(\frac{R_{32}}{R_{33}}\right)\\ \; & \beta =\arctan\left(\frac{-R_{31}}{\sqrt{R_{32}^{2}+R_{33}^{2}}}\right)\\ \; & \gamma =\arctan\left(\frac{R_{21}}{R_{11}}\right) \end{array}$$

where $R_{ij}$ is the value of R at the *i*-th row and the *j*-th column. α, β and γ are the yaw, pitch and roll angle in three degrees of freedom head movement, respectively. The proposed multi-zone template point cloud registration is described as follows.

#### 3.1.1. Multi-Zone Templates Initialization

To reduce reliance on a single template, multiple template point clouds are preset for different gaze zones. These gaze zones are the regions where drivers will commonly allocate glance during driving, and cover the driver’s entire gaze region. The transformation of point cloud registration is dynamically tracked and predicted through particle filtering, as in ref. [6], which ensures the registration accuracy during iterative failure to converge.

For each gaze zone, the template cloud point is obtained and initialized independently. Since the driver’s head is always directly in front of the camera with a clear distance range, the point cloud data of the face area is roughly segmented with simple distance threshold constraints on the generated original point cloud. Nevertheless, the segmentation is not particularly stable by distance, the neck area in particular is easily segmented together, which will affect the cloud point registration accuracy. To segment the head point cloud more accurately, the depth image is pruned and filtered in the restricted region on the detected face area of the RGB image. Its horizontal and vertical region is under the constraint of the proportion among height and width, respectively. Mathematically, $w_{x}$ and $w_{y}$ on the *x* and *y* axis of the depth image can be obtained by: $w_{depth}=\left(f_{x}\cdot w_{rgb}\right)/d_{c}$, $h_{depth}=\left(f_{y}\cdot h_{rgb}\right)/d_{c}$, where, $f_{x}$ and $f_{x}$ are the camera focus parameters at x and y axis, respectively. $d_{c}$ is the face center point value at z axis. $w_{rgb}$ and $h_{rgb}$ are the corresponding location on the RGB image, respectively.

#### 3.1.2. Coarse Transformation via Normal Distributions Transform

When the proposed method cannot effectively predict the current head pose in the initial stage, a normal distributions transform (NDT) [46] is applied to achieve a coarse transformation among the current source and the template from the point cloud data. Based on the head pose conversion formula, the predicted value of the head pose is calculated, and used to obtain the template of the nearest neighbor gaze zone. This predicted value can be used as feedback for updating the parameters of the particle filter. The NDT algorithm is different from the ICP algorithm, in that it performs registration through a probability model of the point cloud. It is fast in efficiency and does not have an over-reliance on the initial values. The coarse transformation via NDT can avoid the registration failure of the ICP algorithm in large head rotation conditions, which ensures the accuracy of the transformation and reduces the effect of noise.

#### 3.1.3. Fine Transformation via ICP Registration

To align two point clouds, the ICP algorithm searches the nearest neighbor point pair between the point clouds, and iteratively calculates the fine transformation matrix by minimizing the least square loss. The noise is smoothed in the point cloud space. The valid nearest neighbor point pairs are respectively obtained in the source point cloud Q and template point cloud P. To optimize the coarse transformation, reconstruction error function is defined and utilized to minimize the transformation. The detailed steps are shown in Algorithm 1. In the right-hand Cartesian coordinate, the head pose can be calculated by the formula via rotation matrix R.


**Algorithm 1:** Multi-zone ICP-based Head Pose Estimation.
**Require:** Multi-gaze-zone cloud point templates ${\left\{P_{m}\right\}}_{m=1}^{M}$, new cloud point Q**Ensure:** Transformation matrix T  1:  Initialize head state by status tracking: $\hat{T}=\left[\begin{array}{cc} \hat{R} & \hat{t}\\ 0 & 1 \end{array}\right]$;  2:  Update coarse head pose by $\alpha =\arctan\left(\frac{{\hat{R}}_{32}}{{\hat{R}}_{33}}\right)$, $\beta =\arctan\left(\frac{-{\hat{R}}_{31}}{\sqrt{{\hat{R}}_{32}^{2}+{\hat{R}}_{33}^{2}}}\right)$, $\gamma =\arctan\left(\frac{{\hat{R}}_{21}}{{\hat{R}}_{11}}\right)$;  3:  Update current gaze zone index *m* by *k*-NN method;  4:  Calculate coarse transformation matrix between Q and $P_{m}$ using Normal Distributions Transform algorithm;  5:  Calculate optimal transformation matrix via ICP algorithm by minimizing reconstruction error:$\left(R,t\right)=\underset{\hat{R},\hat{t}}{\arg\min}\sum_{N_{P}}∥\hat{R}P_{m}+\hat{t}-Q∥$;  6:  **return** Transformation matrix $T=\left[\begin{array}{cc} R & t\\ 0 & 1 \end{array}\right]$;



### 3.2. Multi-Frame Point Cloud Fusion

In a real driving scene, due to insufficient reflection or obstruction of the camera view, the depth image generated by the camera imaging shows many partially missing areas, resulting in the final generated 3D head point cloud data. This causes the registration failure in the single frame point cloud registration. Inspired by Ref. [47], multi-frame point cloud fusion is utilized to obtain more stable head point cloud data through iterative fusion and down-sampling, and finally output the new point cloud. The fused head point cloud can effectively suppress random noise caused by a single frame point cloud.

**Figure 3:**
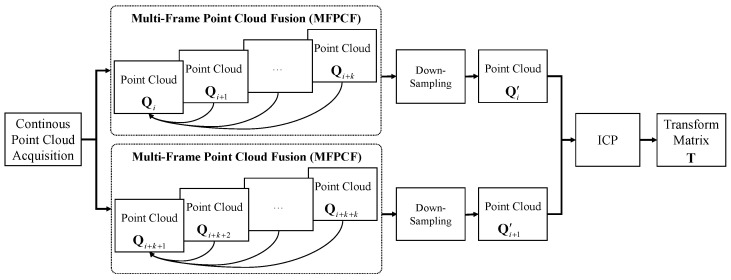
Frame of multi-frame point cloud fusion (MFPCF).

After continuously collecting *K* frame point cloud ${\left\{Q_{k}\right\}}_{k=1}^{K}$, the first frame point cloud $Q_{1}$ is taken as reference point cloud, the second frame point cloud $Q_{2}$ can then be registered to the first frame point cloud. This registration is projected to the same coordinate system according to the transformation matrix, and superimposed into a new point cloud via down-sampling. The later frame point clouds are iterated to form and construct the final point cloud Q′, as shown in Figure 3. In this way, another fused point cloud can be generated through the continuous acquisition of the point cloud. The final transformation matrix T is then computed via the registration of two fused point clouds. Here, the down-sampling of the point cloud is implemented by the farthest point sampling algorithm. The iterative registration will be stopped until the specified target number of point clouds are obtained. The detailed steps are shown in Algorithm 2.


**Algorithm 2:** Multif-frame point cloud fusion and registration.
**Require:** Head region point cloud sequences ${\left\{c_{k}^{1}\right\}}_{k=1}^{K}$ and ${\left\{c_{k}^{2}\right\}}_{k=1}^{K}$**Ensure:** Transformation matrix T  1:Initialization: $Q\leftarrow \left\{c_{1}^{1}\right\}$, $P\leftarrow \left\{c_{1}^{2}\right\}$, $T_{Q}\leftarrow 0$, $T_{P}\leftarrow 0$;  2:
**for** k=2,…,K **do**
  3: Calculate $T_{Q}$ via ICP registration on $\left\{c_{k}^{1}\right\}$ and Q;  4: Transform $\left\{c_{k}^{1}\right\}$ to Q coordinate by $T_{Q}$, and superimposed with Q to form a new point cloud;  5: Down-sampling Q;  6: Calculate $T_{P}$ via ICP registration on $\left\{c_{k}^{2}\right\}$ and P;  7: Transform $\left\{c_{k}^{2}\right\}$ to P coordinate by $T_{P}$, and superimposed with P to form a new point cloud;  8: Down-sampling P;  9:
**end for**
10:Calculate T via ICP registration on P and Q;11:**return** Transformation matrix T;



### 3.3. Eye Image Feature Extractions & Gaze Zone Estimation

This paper uses an SDM landmark detector [48] to locate the face region and eye region, due to its robustness under non-uniform illumination and large head rotation. Similar to Ref. [31], multi-scale spare encoding is adopted for extracting the eye image representation. The eye images are normalized and resized to the same image size. To be specific, the whole eye image is the global scale image, while the partitioned part of the whole eye image is the local scale image. To maintain the important structural information in the image features, the eye images are expressed by the reconstruction parameters with the corresponding spare dictionaries from global and local perspectives. These dictionaries are learned and built by approximate solution. With these obtained dictionaries, the reconstruction parameters of each image are generated by regularized regression. In this paper, the concat parameters of different scales are the related eye image features.

In the real driving environment, the driver’s eye gaze can be replaced by the several gaze zones. At this point, the gaze zone estimation is a classification problem. Thus, a multi-class SVM classifier is utilized to predict the driver’s gaze zone, which combines the eye image features and head pose as input. It contains several binary classifiers with internal nodes and leaves. For each inner classifier, it could be calculated with linear kernel function.

## 4. Experimental Results

This section gives the experimental setup and point cloud collection process to evaluate the proposed method. Based on the experimental data, detailed performance analysis is carried out from two aspects: head pose estimation from point cloud data, and the additional application of gaze zone classification.

### 4.1. Experimental Setup & Data Collection

**Figure 4:**
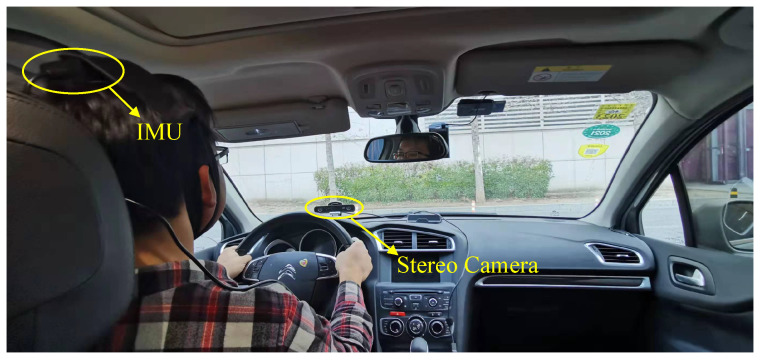
Setup of data acquisition in the real driving environment.

**Figure 5:**
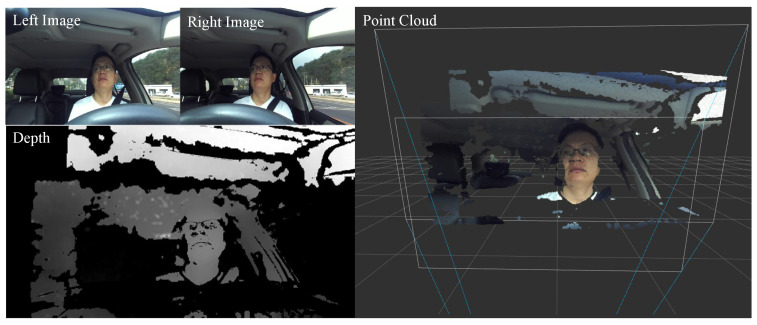
Examples obtained by the stereo camera.

To record point cloud data, a ZED2 stereo camera was used to collect the driver’s face video in the real driving conditions. The ZED2 camera has a compact appearance and was equipped with two wide-angle lenses for a reachable field of view. The best applicable range of the ZED2 camera is 0.5∼20 m. In this paper, it was mounted on the underside of the windshield and directly in front of the driver (as shown in Figure 4), where it does not interfere with the front field of view of the driver, and can also meet the camera’s collection requirements. The image resolution is 720 P (the stereo image size is 2560×720, while the monocular image size is 1080×720). The sampling rate of the camera is set to 60 FPS, to ensure the driver’s head can be captured continuously in the driving scene even if the driver’s head moves quickly. As shown in Figure 5, the upper left is the example RGB image, and the bottom left is the corresponding example depth image. The right is the point cloud generated after image calibration. It is roughly segmented by simple distance threshold constraints on the original point cloud data.

An IMU sensor was mounted on the driver’s head, and connected to the laptop through a USB to TTL serial module. The IMU had a built-in Kalman filter, which can directly output the three-axis Euler angle, with a static accuracy of 0.05 degree, and the frequency is set to 200 HZ. The output value of the IMU sensor was calibrated and then used as the ground-truth value of the head pose.

**Figure 6:**
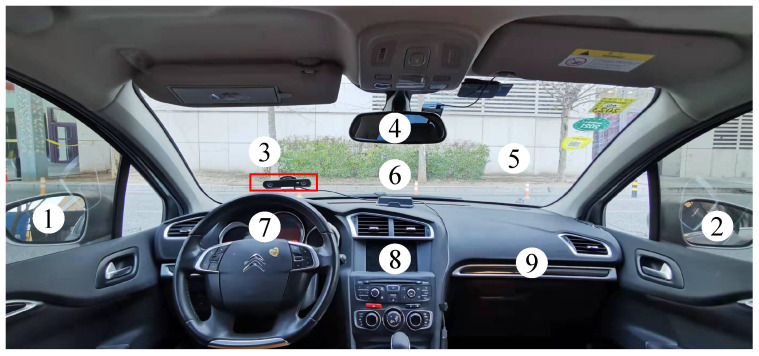
Gaze zone partition in the naturalistic driving condition.

We divided the gaze region in front of the driver into several gaze zones, which basically covers most of the gaze region that needs to be looked at during normal driving. The nine gaze zones are windshield left-side (forward-view zone), left-side mirror, right-side mirror, rear-view mirror, instrument panel, center console, windshield center, windshield right-side, and glove box, as shown in Figure 6. The centre point of the forward-view zone (gaze zone #3) is set as the origin of the head pose, its anchor point is $0^{\circ }$ in the three-dimensional Euler angle (yaw, pitch, and roll). Before continuously collecting the gaze data, the driver was asked to face the center of each gaze zone in turn to generate the point cloud templates and calibrate anchor points of the gaze zone. For each gaze zone, 60 frames of data were recorded continuously, with one second fixation. A stable point cloud template was formed based on the first reference frame, and superimposed sequentially with the following 59 frames point cloud data at a 50% down-sampling rate. For some gaze zone templates, the partial occlusion caused by the reflection might still be exited, especially the gaze zone with large head rotation.

A total of 40,000 valid frame data points are collected for evaluation. The accuracy of the gyroscope is high enough to be used as the ground-truth value for head pose tracking in the directions of yaw, pitch, and roll. Since the acquisition frequency of the camera and the gyroscope were inconsistent, the data were aligned with the timeline. Table 1 shows the head rotation range of the evaluation dataset. In addition, the labels of the gaze zone were not only generated by the nearest neighbor classifier automatically, but also corrected by the participant manually.

### 4.2. Evaluations on Head Pose Tracking

This section experiments with the proposed head pose tracking method. The estimation accuracy of the three directions (yaw, pitch, and roll) is first verified. Then the estimated accuracy is compared and analyzed in each gaze zone. Next, the proposed method is compared with other baseline methods.

#### 4.2.1. Comparison on Yaw, Pitch and Roll

**Figure 7:**
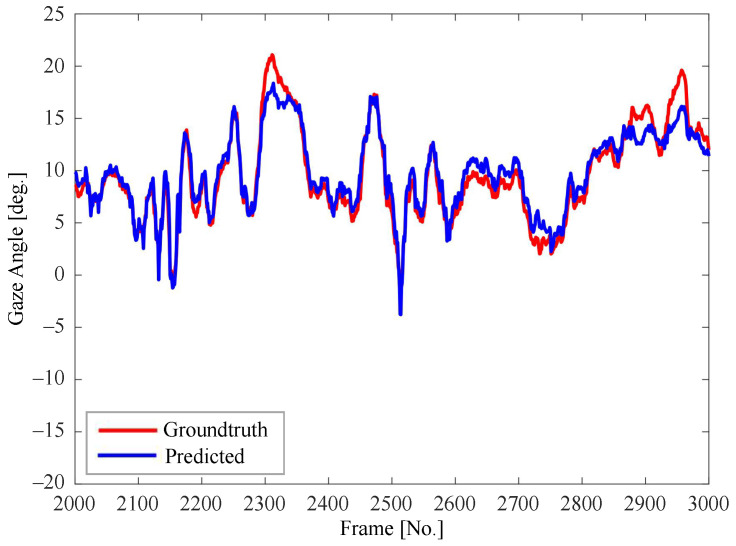
Comparison between the predicted value and the ground-truth value of yaw angle.

**Figure 8:**
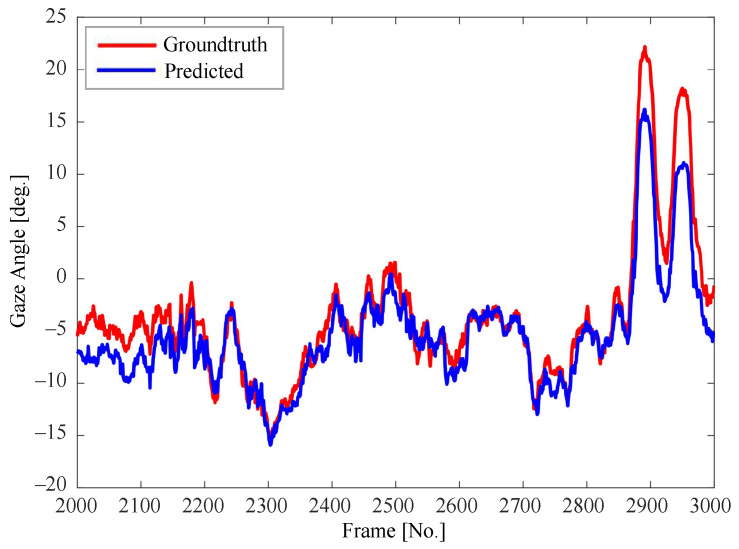
Comparison between the predicted value and the ground-truth value of pitch angle.

**Figure 9:**
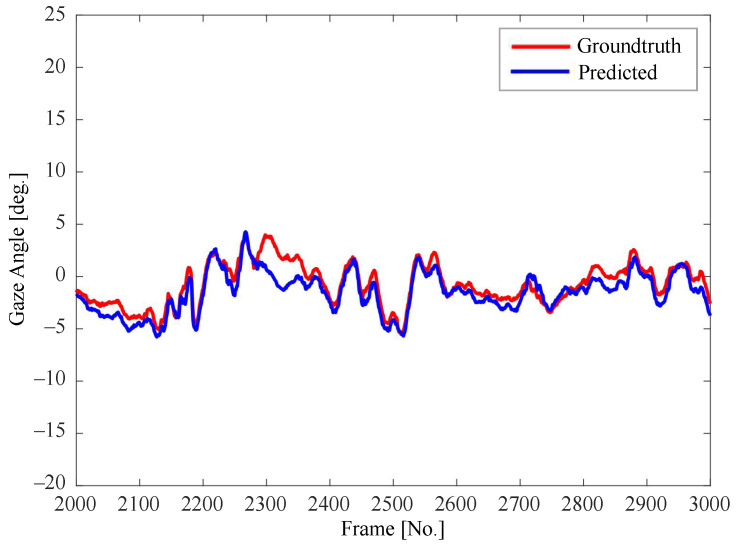
Comparison between the predicted value and the ground-truth value of roll angle.

Figure 7, Figure 8 and Figure 9 show the consecutive estimation results of 1000 frames randomly selected in the yaw, roll and pitch directions, respectively. The predicted head pose and ground-truth head pose are denoted in red lines and blue lines, respectively. The predicted values in all directions are basically consistent with the trends of the ground-truth values.

**Figure 10:**
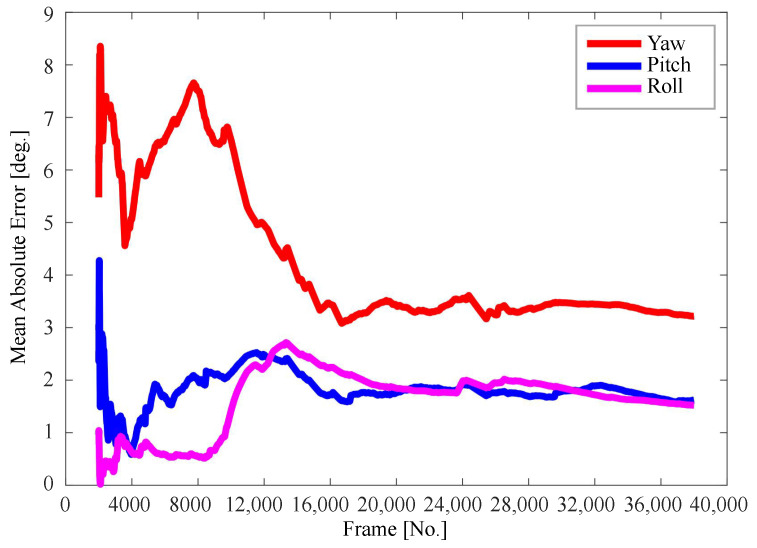
Mean absolute error curve in the yaw, pitch and roll directions.

Figure 10 shows the mean absolute error curve of the entire dataset in the directions of yaw, roll, and pitch. It can be seen that the accumulated errors in the directions of yaw, pitch, and roll are descending sorted. The yaw error is obviously larger than that of the other two directions. One possible reason is that the head rotation in the yaw direction is extremely large in the naturalistic driving condition.

#### 4.2.2. Comparison on Gaze Zones

Taking the ground-truth value of the IMU as a reference, the average estimation error in a certain range of each gaze zone is compared and analyzed. Since the horizontal movement of the head pose is large, the range of the yaw angle is relatively larger than the range of the pitch angle and roll angle when adjusting posture. Here, the tolerance threshold is set to 5 degrees in the Yaw direction, and 2 degrees in the other two directions.

Table 2 gives the mean absolute error value of head pose estimation in the directions of yaw, pitch, and roll for each gaze zone. It can be seen that the estimation error is large on gaze zone #2. This gaze zone is far away from the driver, and the head rotation is large. Among all gaze zones, the one with the smallest comprehensive error in the three directions is the front gaze zone (gaze zone #3). The gaze zone corresponding to the smallest head movement amplitude has more high quality point cloud data, and a better ICP registration performance.

#### 4.2.3. Comparison with Baseline Methods

Several baseline methods are used to compare and analyze the performance of head pose estimation, such as conventional methods (POSIT [49], García et al. [8]), deep learning-based methods (PointNetLK [20], Hu et al. [10]), and opensource tools (OpenFace 2.0 [50]). To evaluate the effect of the point cloud fusion, results without the multi-frame point cloud fusion (MFPCF) are also performed. The ICP-based head pose tracking was solved by removing the fusion processing, and matching directly on the point cloud data. The deep learning-based methods were tested on a computer with Intel i7-6700k CPU, 32 GB RAM, and NVIDIA GeForce GTX 980 Ti GPU. To be clear, the method of Hu et al. did not work correctly on our dataset, so here the results are taken from Ref. [10].

Table 3 illustrates the mean absolute error value of different methods. The ablation study shows that the proposed method has an important compensation effect on the data with large head rotation. The accuracy of the PointNetLK method is the lowest. In most cases, it cannot obtain valid results. This may be caused by the inability of the method to extract features on the point cloud with large occlusion. Due to the lack of head position information, the pre-set parameters are not adapted to the POSIT model. OpenFace 2.0 has a large estimation error under large rotation, and its prediction on pitch and roll directions is not stable. Compared with the baseline methods, the estimation results of the proposed method have better accuracy, especially in the yaw direction.

### 4.3. Evaluations on Gaze Zone Estimation

**Figure 11:**
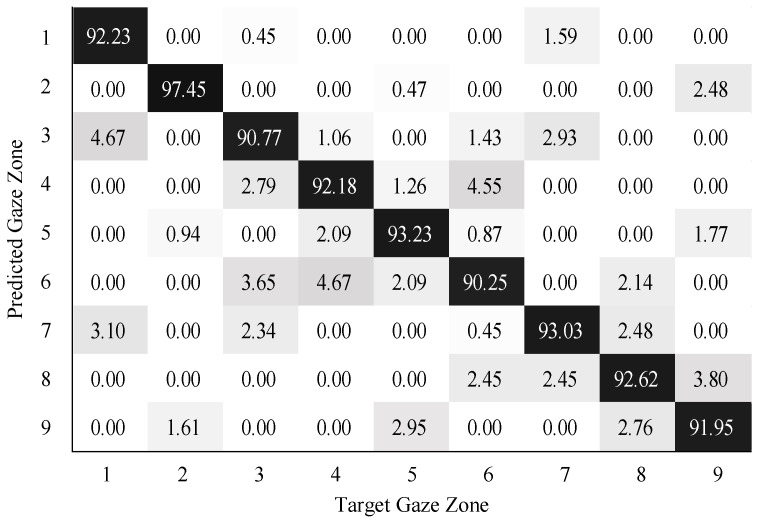
Gaze zone classification results based on the POSIT method and SVM classifier.

**Figure 12:**
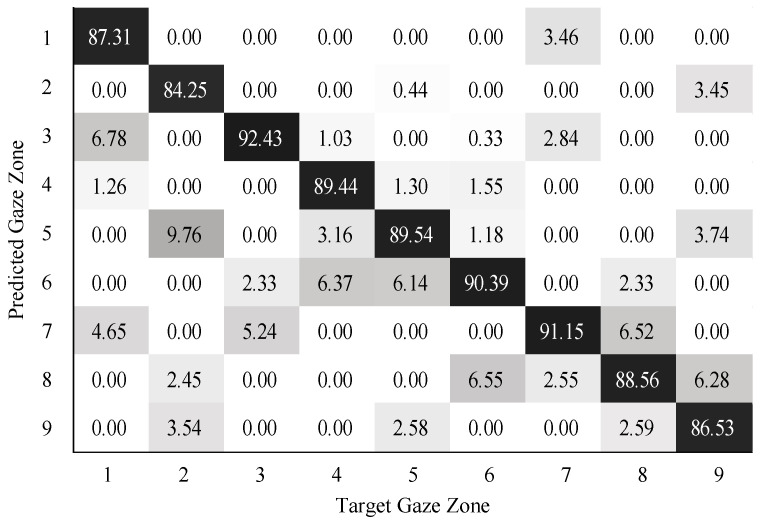
Gaze zone classification results based on the PointNetLK method and SVM classifier.

**Figure 13:**
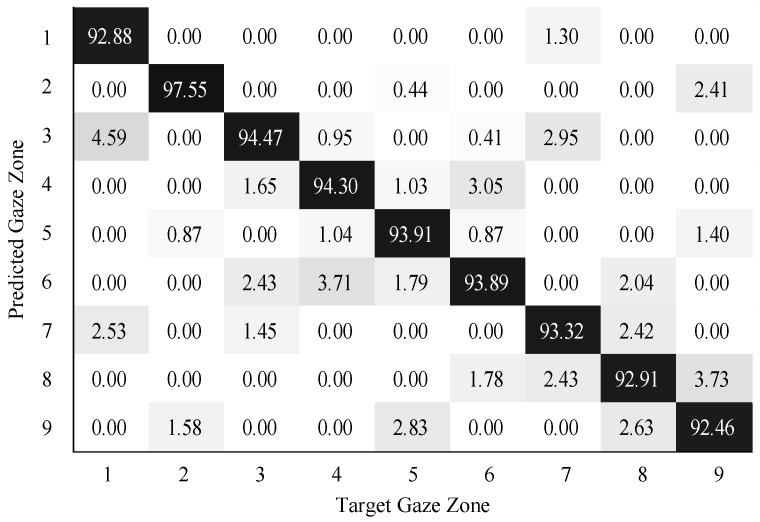
Gaze zone classification results based on the proposed method and SVM classifier.

**Figure 14:**
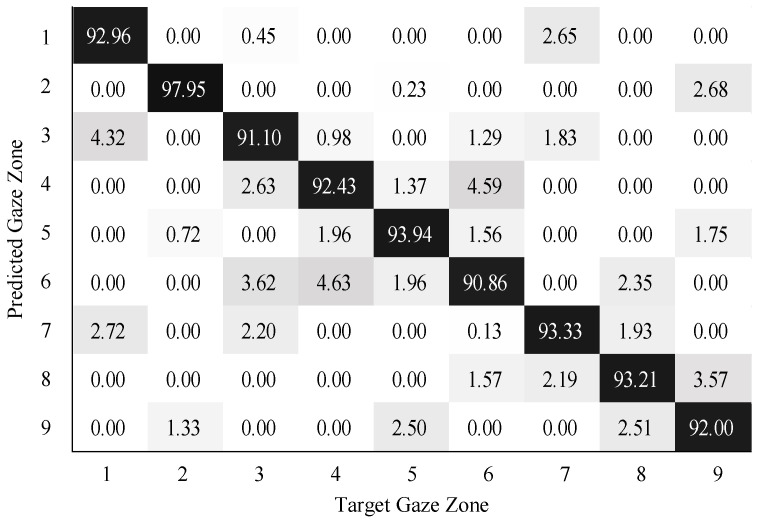
Gaze zone classification results based on POSIT method and NN classifier.

**Figure 15:**
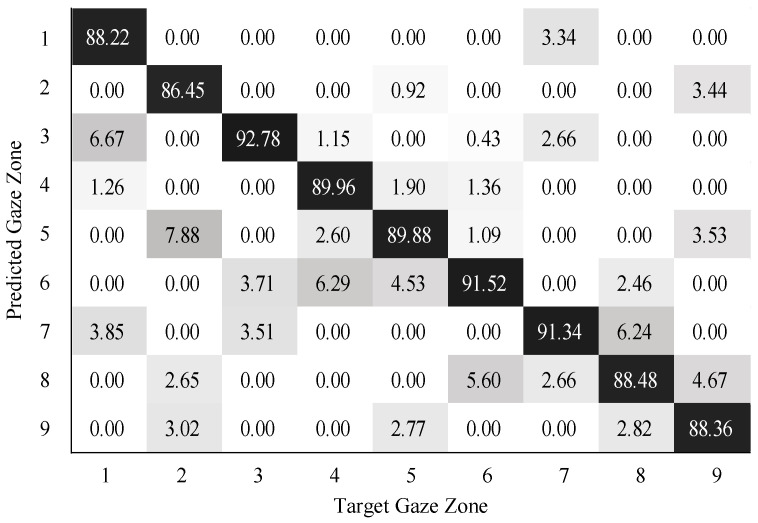
Gaze zone classification results based on the PointNetLK method and NN classifier.

**Figure 16:**
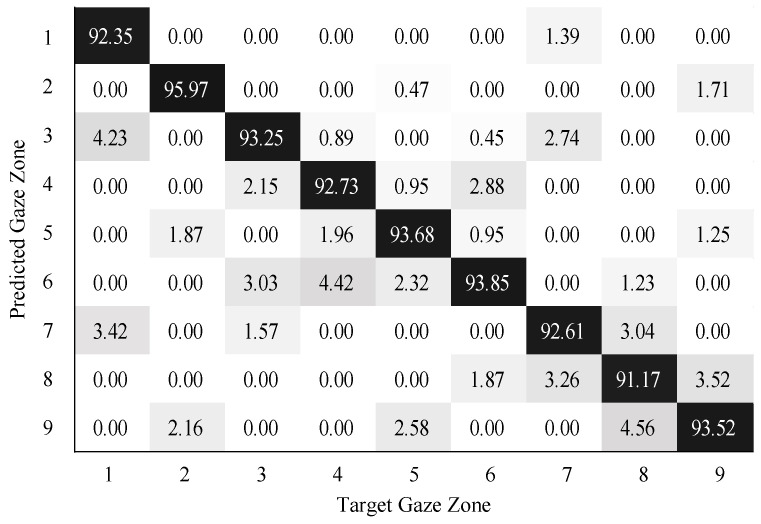
Gaze zone classification results based on the proposed method and NN classifier.

To validate the performance of gaze zone estimation, the dataset was separated into a training dataset and a testing dataset. The training dataset was made up of 30,000 labeled data points randomly selected from the original dataset, and the remaining 10,000 labeled data points were then used as testing data. For all the RGB images, the face and eye regions were segmented via facial landmarks detection. The eye images were intercepted based on the locations of eye regions. The uniform size of the eye image was 80×40. According to the eye image representation, a global sparse dictionary with 1024 bases and a local sparse dictionary with 25 bases were constructed for encoding and decoding the eye image. The global size of the eye images is the same as the eye image resolution, the patch size of the eye images is 10×10 in blocks. The SVM classifier was trained on the head features and eye image features. The neural networks (NN) classifier with softmax was trained in a similar way as the SVM classifier. The confusion matrix results on gaze zone classification with the SVM classifier are shown in Figure 11, Figure 12 and Figure 13. The confusion matrix results with the NN classifier are shown in Figure 14, Figure 15 and Figure 16. Here, the classifiers with the head pose vector predicted by POSIT, PointNetLK, and our method, were chosen for model training, respectively.

The average gaze zone estimation accuracy of the SVM classifier with the POSIT method, the PointNetLK method, and the proposed method is 92.63%, 88.84%, and 93.97%, respectively. While the average gaze zone estimation accuracy of the NN classifier with the POSIT method, the PointNetLK method, and the proposed method is 93.09%, 89.67%, and 93.24%, respectively. It is worth noting that the classification of the method has better accuracy in dense gaze zones, such as gaze zone #3, gaze zone #4, and gaze zone #6. The classification accuracy is not much different among other gaze zones. The POSIT method results in large estimation errors in the adjacent gaze zones at the windshield. Since the POSIT method is based on the average head model and the weak perspective assumptions, misjudgments occurred in the gaze zone classification due to the lack of spatial position information of the head feature points. The PointNetLK method obtains invalid values in several non-frontal gaze zones. This paper only performs the gaze zone classification on the valid data of the PointNetLK method. It can be seen that the gaze zones with large pitch and roll directions are easily misestimated.

## 5. Conclusions

In this paper, a novel driver head pose and gaze zone estimation method is proposed by using an RGB-D camera. This paper uses multi-frame point cloud fusion to generate a stable point cloud. The fused point cloud is then used to calculate the best transformation of the registration. Multi-zone templates are adopted to quickly locate the nearest neighbor point cloud template. At the same time, the method of tracking and predicting the initial coarse transformation based on a particle filter and normal distributions transform is studied, which improves the efficiency and accuracy of point cloud registration. The experimental results demonstrate that the proposed method can achieve accurate results on both head pose tracking and gaze zone classification.

## Figures and Tables

**Table 1:** Dataset head pose range.

Head Pose	Maximum (${}^{\circ }$)	Minimum (${}^{\circ }$)	Data Range (${}^{\circ }$)
Yaw	50	−38	88
Pitch	25	−20	45
Roll	10	−10	20

**Table 2:** Mean absolute error of head pose estimation in each gaze zone.

Gaze Zone	Yaw (${}^{\circ }$)	Pitch (${}^{\circ }$)	Roll (${}^{\circ }$)
1	3.48	1.57	1.57
2	3.47	1.72	1.63
3	2.88	1.16	1.10
4	3.15	1.67	1.58
5	3.09	1.58	1.62
6	3.02	1.61	1.05
7	3.05	1.35	1.49
8	3.35	1.65	1.42
9	3.40	1.77	1.51

**Table 3:** Comparison of different head pose estimation methods.

Method	Yaw (${}^{\circ }$)	Pitch (${}^{\circ }$)	Roll (${}^{\circ }$)
OpenFace 2.0 [50]	5.71	5.54	6.16
POSIT [49]	5.65	3.26	2.94
García et al. [8]	3.70	2.10	2.90
PointNetLK [20]	7.61	8.76	4.38
Hu et al. [10]	7.32	6.68	5.91
Our method (without MFPCF)	4.49	1.93	1.85
Our method	3.37	1.61	1.52

## Data Availability

Not applicable.

## References

[1] Kaplan S., Guvensan M.A., Yavuz A.G., Karalurt Y. (2015). Driver behavior analysis for safe driving: A survey. IEEE Trans. Intell. Transp. Syst..

[2] Mittal A., Kumar K., Dhamija S., Kaur M. Head movement-based driver drowsiness detection: A review of state-of-art techniques. Proceedings of the 2016 IEEE International Conference on Engineering and Technology (ICETECH).

[3] Wang J., Chai W., Venkatachalapathy A., Tan K.L., Haghighat A., Velipasalar S., Adu-Gyamfi Y., Sharma A. (2021). A Survey on Driver Behavior Analysis from In-Vehicle Cameras. IEEE Trans. Intell. Transp. Syst..

[4] Fanelli G., Gall J., Van Gool L. Real time head pose estimation with random regression forests. Proceedings of the CVPR 2011.

[5] Zhang Z., Lian D., Gao S. (2021). RGB-D-based gaze point estimation via multi-column CNNs and facial landmarks global optimization. Vis. Comput..

[6] Wang Y., Yuan G., Mi Z., Peng J., Ding X., Liang Z., Fu X. (2019). Continuous driver’s gaze zone estimation using rgb-d camera. Sensors.

[7] Meyer G.P., Gupta S., Frosio I., Reddy D., Kautz J. Robust model-based 3d head pose estimation. Proceedings of the IEEE international Conference on Computer Vision.

[8] Peláez C.G.A., García F., de la Escalera A., Armingol J.M. (2014). Driver monitoring based on low-cost 3-D sensors. IEEE Trans. Intell. Transp. Syst..

[9] Bär T., Reuter J.F., Zöllner J.M. Driver head pose and gaze estimation based on multi-template icp 3-d point cloud alignment. Proceedings of the 2012 15th International IEEE Conference on Intelligent Transportation Systems.

[10] Hu T., Jha S., Busso C. Robust driver head pose estimation in naturalistic conditions from point-cloud data. Proceedings of the 2020 IEEE Intelligent Vehicles Symposium (IV).

[11] Hu T., Jha S., Busso C. (2021). Temporal head pose estimation from point cloud in naturalistic driving conditions. IEEE Trans. Intell. Transp. Syst..

[12] Huang X., Mei G., Zhang J., Abbas R. (2021). A comprehensive survey on point cloud registration. arXiv.

[13] Cheng L., Chen S., Liu X., Xu H., Wu Y., Li M., Chen Y. (2018). Registration of laser scanning point clouds: A review. Sensors.

[14] Padeleris P., Zabulis X., Argyros A.A. Head pose estimation on depth data based on particle swarm optimization. Proceedings of the 2012 IEEE Computer Society Conference on Computer Vision and Pattern Recognition Workshops.

[15] Schwarz A., Haurilet M., Martinez M., Stiefelhagen R. Driveahead-a large-scale driver head pose dataset. Proceedings of the IEEE Conference on Computer Vision and Pattern Recognition Workshops.

[16] Borghi G., Venturelli M., Vezzani R., Cucchiara R. Poseidon: Face-from-depth for driver pose estimation. Proceedings of the IEEE Conference on Computer Vision and Pattern Recognition.

[17] Venturelli M., Borghi G., Vezzani R., Cucchiara R. (2016). Deep head pose estimation from depth data for in-car automotive applications. Proceedings of the International Workshop on Understanding Human Activities through 3D Sensors.

[18] Saeed A., Al-Hamadi A. Boosted human head pose estimation using kinect camera. Proceedings of the 2015 IEEE International Conference on Image Processing (ICIP).

[19] Ribeiro R.F., Costa P.D. Driver gaze zone dataset with depth data. Proceedings of the 2019 14th IEEE International Conference on Automatic Face & Gesture Recognition (FG 2019).

[20] Aoki Y., Goforth H., Srivatsan R.A., Lucey S. Pointnetlk: Robust & efficient point cloud registration using pointnet. Proceedings of the IEEE/CVF Conference on Computer Vision and Pattern Recognition.

[21] Qi C.R., Su H., Mo K., Guibas L.J. Pointnet: Deep learning on point sets for 3d classification and segmentation. Proceedings of the IEEE Conference on Computer Vision and Pattern Recognition.

[22] Huang X., Mei G., Zhang J. Feature-metric registration: A fast semi-supervised approach for robust point cloud registration without correspondences. Proceedings of the IEEE/CVF Conference on Computer Vision and Pattern Recognition.

[23] Qi C.R., Yi L., Su H., Guibas L.J. Pointnet++: Deep hierarchical feature learning on point sets in a metric space. Proceedings of the Advances in Neural Information Processing Systems.

[24] Chen Y., Medioni G. (1992). Object modelling by registration of multiple range images. Image Vis. Comput..

[25] Besl P.J., McKay N.D. Method for registration of 3-D shapes. Proceedings of the Sensor Fusion IV: Control Paradigms and Data Structures, SPIE.

[26] Yang J., Li H., Jia Y. Go-icp: Solving 3d registration efficiently and globally optimally. Proceedings of the IEEE International Conference on Computer Vision.

[27] Pavlov A.L., Ovchinnikov G.W., Derbyshev D.Y., Tsetserukou D., Oseledets I.V. AA-ICP: Iterative closest point with Anderson acceleration. Proceedings of the 2018 IEEE International Conference on Robotics and Automation (ICRA).

[28] Jha S., Busso C. Challenges in head pose estimation of drivers in naturalistic recordings using existing tools. Proceedings of the 2017 IEEE 20th International Conference on Intelligent Transportation Systems (ITSC).

[29] Fridman L., Langhans P., Lee J., Reimer B. (2016). Driver gaze region estimation without use of eye movement. IEEE Intell. Syst..

[30] Wang Y., Zhao T., Ding X., Bian J., Fu X. Head pose-free eye gaze prediction for driver attention study. Proceedings of the 2017 IEEE International Conference on Big Data and Smart Computing (BigComp).

[31] Yuan G., Wang Y., Peng J., Fu X. (2021). A Novel Driving Behavior Learning and Visualization Method with Natural Gaze Prediction. IEEE Access.

[32] Tayibnapis I.R., Choi M.K., Kwon S. Driver’s gaze zone estimation by transfer learning. Proceedings of the 2018 IEEE International Conference on Consumer Electronics (ICCE).

[33] Bi Q., Ji X., Sun Y. Research on Driver’s Gaze Zone Estimation Based on Transfer Learning. Proceedings of the 2020 IEEE International Conference on Information Technology, Big Data and Artificial Intelligence (ICIBA).

[34] Shehu I.S., Wang Y., Athuman A.M., Fu X. (2021). Remote Eye Gaze Tracking Research: A Comparative Evaluation on Past and Recent Progress. Electronics.

[35] Khan M.Q., Lee S. (2019). Gaze and eye tracking: Techniques and applications in ADAS. Sensors.

[36] Wang Y., Shen T., Yuan G., Bian J., Fu X. (2016). Appearance-based gaze estimation using deep features and random forest regression. Knowl.-Based Syst..

[37] Wang Y., Zhao T., Ding X., Peng J., Bian J., Fu X. (2018). Learning a gaze estimator with neighbor selection from large-scale synthetic eye images. Knowl.-Based Syst..

[38] Lundgren M., Hammarstrand L., McKelvey T. (2016). Driver-gaze zone estimation using Bayesian filtering and Gaussian processes. IEEE Trans. Intell. Transp. Syst..

[39] Yuan G., Wang Y., Yan H., Fu X. (2022). Self-calibrated driver gaze estimation via gaze pattern learning. Knowl.-Based Syst..

[40] Jha S., Busso C. (2020). Estimation of Driver’s Gaze Region from Head Position and Orientation using Probabilistic Confidence Regions. arXiv.

[41] Fridman L., Lee J., Reimer B., Victor T. (2016). ‘Owl’and ‘Lizard’: Patterns of head pose and eye pose in driver gaze classification. IET Comput. Vis..

[42] Ledezma A., Zamora V., Sipele Ó., Sesmero M.P., Sanchis A. (2021). Implementing a Gaze Tracking Algorithm for Improving Advanced Driver Assistance Systems. Electronics.

[43] Araluce J., Bergasa L.M., Ocaña M., López-Guillén E., Revenga P.A., Arango J.F., Pérez O. (2021). Gaze Focalization System for Driving Applications Using OpenFace 2.0 Toolkit with NARMAX Algorithm in Accidental Scenarios. Sensors.

[44] Chiou C.Y., Wang W.C., Lu S.C., Huang C.R., Chung P.C., Lai Y.Y. (2019). Driver monitoring using sparse representation with part-based temporal face descriptors. IEEE Trans. Intell. Transp. Syst..

[45] Yang Y., Liu C., Chang F., Lu Y., Liu H. (2021). Driver Gaze Zone Estimation via Head Pose Fusion Assisted Supervision and Eye Region Weighted Encoding. IEEE Trans. Consum. Electron..

[46] Magnusson M., Andreasson H., Nuchter A., Lilienthal A.J. Appearance-based loop detection from 3D laser data using the normal distributions transform. Proceedings of the 2009 IEEE International Conference on Robotics and Automation.

[47] Li S., Ngan K.N., Paramesran R., Sheng L. (2015). Real-time head pose tracking with online face template reconstruction. IEEE Trans. Pattern Anal. Mach. Intell..

[48] Vicente F., Huang Z., Xiong X., De la Torre F., Zhang W., Levi D. (2015). Driver gaze tracking and eyes off the road detection system. IEEE Trans. Intell. Transp. Syst..

[49] Martins P., Batista J. Accurate single view model-based head pose estimation. Proceedings of the 2008 8th IEEE International Conference on Automatic Face & Gesture Recognition.

[50] Baltrusaitis T., Zadeh A., Lim Y.C., Morency L.P. Openface 2.0: Facial behavior analysis toolkit. Proceedings of the 2018 13th IEEE International Conference on Automatic Face & Gesture Recognition (FG 2018).

